# Modification of the Structure and Properties of Lightweight Cement Composite with PVA Fibers

**DOI:** 10.3390/ma14205983

**Published:** 2021-10-11

**Authors:** Donatas Sikarskas, Valentin Antonovič, Jurgita Malaiškienė, Renata Boris, Rimvydas Stonys, Genadijs Šahmenko

**Affiliations:** 1 Laboratory of Composite Materials, Vilnius Gediminas Technical University, LT-10223 Vilnius, Lithuania; donatas.sikarskas@vilniustech.lt (D.S.); valentin.antonovic@vilniustech.lt (V.A.); renata.boris@vilniustech.lt (R.B.); rimvydas.stonys@vilniustech.lt (R.S.); 2 Institute of Materials and Structures, Riga Technical University, LV-1048 Riga, Latvia; genadijs.sahmenko@rtu.lv

**Keywords:** lightweight composite, granulated expanded glass, polyvinyl alcohol fibers, microstructure, physical-mechanical properties

## Abstract

This study addresses the application of polyvinyl alcohol (PVA) fibers to improve the performance of lightweight cement composites with pozzolans. Blended cement mixes based on expanded glass granules were modified with PVA fibers (Type A: Ø40 µm, L = 8 mm and Type B: Ø200 µm, L = 12 mm). The following research methods were used to analyse the effect of the fibers on the structure of cement matrix and physical-mechanical properties of lightweight composite: SEM, XRD, DTG, calorimetry tests, and standard test methods of physical and mechanical properties. Results from the tests showed that a denser layer of hydrates was formed around the PVA fiber and the amounts of portlandite, CSH, and CASH formed in the specimens with PVA were found to be higher. PVA fibers of Type A accelerated hydration of the cement paste, slightly increased the compressive strength of the lightweight composite, but had no significant effect on the values of density, ultrasonic pulse velocity and flexural strength. The shrinkage of cement composite was significantly reduced using both types of PVA fiber and both types of PVA fibers increased the fracture energy of lightweight cement composite with expanded granules.

## 1. Introduction

Polyvinyl alcohol (PVA) fibers were found to significantly improve the ductility, fracture toughness and impact resistance of lightweight concrete [1,2]. They strengthen the surface bonds between the aggregate, matrix and reinforcement and also reduce the formation of flocculated structures by cement particles and the bleeding effect of cement paste, improves workability [1,2,3]. The enhanced ductility of cement composite depends on the characteristics of the matrix and the fibers as well as on the appropriate amount of reinforcement, so that the load originally carried by the matrix would be transferred to the bridging fibers [4]. The paper [5] reported that 0.5–0.75% of polymer microfibers is the most effective content for improving the properties of cement-based concrete. According to the literature review, fibers must have a much higher modulus of elasticity in order to increase the strength of concrete; however, this property is often difficult to achieve because the modulus of elasticity of cement-based matrices ranges from 15 GPa to 30 GPa.

PVA fibers with a high modulus of elasticity can reduce the crack width and thus are the most suitable for reducing the drying shrinkage, which is one of the most important characteristics of lightweight composites [6,7]. The shrinkage of the composite is limited physically by a strong adhesion between the fibre and the cement matrix [8,9]; besides, the fibres increase the tensile strength of the composite, which also reduces shrinkage and potential cracking.

PVA fibers can change the pattern of water movement in the cementitious matrix by attracting more water to the surface of the fibers [10]. An extremely strong bond between the fibers and the cement matrix in the conventional cement mix is formed due to the hydrophilic character of PVA fibers. It was determined that a denser CSH layer is formed around PVA fibers of a certain size. The denser is this layer, the stronger is the connection between PVA fibers and cement matrix [11,12,13,14,15]. It was established [15] that pozzolanic additives (especially nano SiO_2_) accelerated cement hydration and contributed to the formation of additional CSH, which covered the surface of the PVA fibers. The CSH layer improved the bonding between PVA fibers and cement-based matrix and resulted in an efficient load-transfer by fibre bridging. Researchers [16,17,18,19,20,21] have found that a higher amount of CSH produced in the pozzolanic reaction reduces the porosity of the cement composite and at the same time reduces the drying shrinkage. Spent fluid catalytic cracking catalyst waste (FCCCW) from oil refineries, containing silica and alumina, accelerate cement hydration while the pozzolanic reactions have a positive effect on the mechanical characteristics of hardened cement paste [22,23,24,25]. Metakaolin waste MW generated in the manufacture of granulated expanded glass [26] consists of metakaolin and glass waste. It has pozzolanic activity and also improves the mechanical properties of the material [27]. Our previous research work [10] revealed that cement hydration can be controlled and mechanical properties of lightweight composite materials can be improved through the combined use of FCCCW, which accelerates the hydration of Portland cement, and MW addition, which has a retarding effect. On the other hand, an excessively strong a chemical bond between PVA and cementitious hydrates can reduce the elasticity of the composite, cause the formation of cracks, and premature breakage of the fibers [13,14].

Agglomeration of the fibers may also reduce it positive effect. The strength decreases at the higher content of fibers [28] because the fibers entrap air voids during mixing and increase the porosity. The increase in porosity depends on the amount, shape and length of the fibers. Besides the addition of fibers deteriorates the workability of the cement paste, make its harder to compact and increase porosity.

Taking into account the above aspects it is very important to choose the appropriate amount and type of PVA fibers. The quality of the cementitious binder, which is greatly influenced by pozzolanic additives, and its interaction with PVA fiber reinforcement, can have a significant impact on the properties of lightweight aggregate composite. Granulated expanded glass (GEG) used in combination with pozzolanic additives in cement-based materials is expected not only to improve their mechanical properties but also to mitigate alkali aggregate reactions, increase their durability [29,30]. Lightweight plaster with GEG can replace dangerous asbestos in the masonry structures of energy units or can be used in fire resistance refractory mortars applied to protect metal structures in chemical plants. GEG can be also used in refractory mortar for the insulation of the interior of energy units because light composite materials containing GEG retain heat, sustain mechanical impact and resist deformation in the temperature up to 600 °C. The basis for the development of such a composite is low density and good mechanical characteristics of GEG [31]. It also has good frost resistance properties, because the porous structure contributes to decreasing the microdamage caused by the crystallization of ice [31]. It should also be noted that glass-based filler is sufficiently resistant to alkaline corrosion because the gel formed during the alkali-aggregate reaction penetrates into the granules without creating high stresses [31,32,33].

The aim of this study was to determine the effect of PVA fibers of different types (Type A: Ø40 µm, L = 8 mm and Type B: Ø200 µm, L = 12 mm) on the hydration and structure of the cement matrix modified with combined pozzolanic additive and physical-mechanical properties of the lightweight composite containing GEG.

## 2. Materials and Methods

The chemical analysis of raw materials was determined with a ZSX Primus IV wavelength dispersive X-ray fluorescence spectrometer (XRF, Rigaku, Tokyo, Japan) (X-ray tube, Rh-anode, 4 kW, 60 kV, specimen size 37 mm (diameter)). The tablets of powders (<50 µm) were prepared by using a 20 t force hydraulic press.

Portland cement (PC) CEM I 42.5 R (Rocket cement M-600, Heidelberg Cement, Skovde, Sweden) was used for the tests. The chemical composition of the cement is presented in Table 1. Mineral composition of the cement used: C_3_S—56.6%, C_2_S—16.7%, C_3_A—9.0%, C_4_AF—10.6% and 7.2% of other substances (alkaline sulphates and CaO).

The chemical composition of spent fluidized bed catalytic cracking catalyst waste (AB Orlen Lietuva oil refinery, Mazeikiai, Lithuania) and metakaolin based waste MW (UAB Stikloporas, Druskininkai, Lithuania) is presented in Table 2. FCCCW particles are spherical, with an average diameter of ~40 μm [34].

MW is generated in the manufacturing of granulated expanded glass. It is a mix of metakaolin and residues of granules. GEG of three different fractions (1.0–2.0 mm, 0.5–1.0 mm and 0.25–0.5 mm) were used as lightweight composite aggregates. GEG characteristics are presented in Table 3.

PVA fibers are superficially treated to have an optimal adhesion with the interfacing cement matrix. The fibers have a specific weight of 1300 kg/m^3^, other characteristics are presented in Table 4.

**Figure 1:**
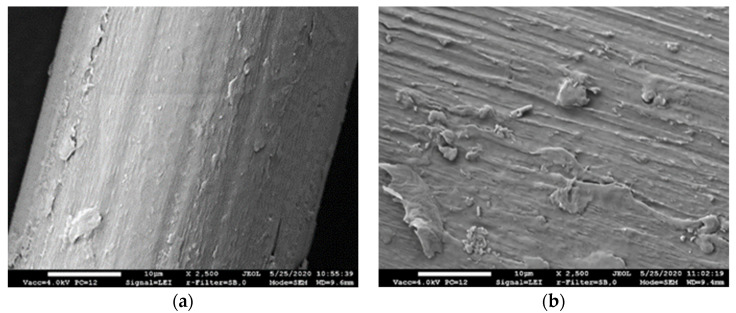
Surface of PVA fiber: (**a**) PVA fiber of Type A; (**b**) PVA fiber of Type B.

SEM images of Type A and Type B fibers used are presented in Figure 1. The images show that the fibers of both types have uneven rough surfaces with visible grooves with a width of 1–3 µm.

The superplasticizer Melment F10 (SP) (BASF Construction Polymers GmbH, Trotsberg, Germany) used for the mixtures is a free-flowing spray dried powder of a sulphonated polycondensation product based on melamine. It has a pH value of 9.4 at 20 °C temperature in 20% solution. Tap water was used for the mixtures.

Compositions of cement paste for XRD, DTG, MIP analysis and lightweight composite used in the tests described in this paper are presented in Table 5.

Specimens of blended cement paste and lightweight cement-based composition were mixed in a Hobart Type mixer for 2 min in dry stirring and for two more minutes after adding water. Specimens of 160 × 40 × 40 mm size were formed on the vibration table. The drying shrinkage deformation of lightweight composite was measured from the second day of curing until the 28th day. The specimens were cured for 24 h in moulds at 20 ± 2 °C and at least 90% relative humidity. On the second day they were demoulded and cured for 27 days at 20 ± 2 °C temperature and 65 ± 5% relative humidity.

The density was calculated according to specimen weight (accuracy 0.01 g) and the volume was determined by dimensions (accuracy 0.01 mm).

Ultrasonic pulse velocity (UPV) was measured by direct transmission method using a Pundit 7 device equipped with two 54 kHz transducers (transmitter and receiver) firmly coupled to the farthest opposite ends of the paste prims using petroleum jelly as the couplant between the transducers and the specimens. The UPV was calculated using Equation (1) (UPV, m/s):

UPV = l/τ(1)

where: l is the length of ultrasonic pulse path through the specimen, m/s i.e., the distance between the two transducers (0.16 m); τ is signal propagation time provided by the test equipment, s.

6 specimens of each batch of lightweight composite were used to determine the physical-mechanical properties: 3 of them were used to determine the mechanical properties at 7 days of curing and 3 at 28 days. The specimens were moulded, cured and hardened according to the standard LST EN 1015-11:2007. The flexural and compressive strength of the specimens was measured by an ALPHA3-3000S hydraulic press (Form+Test Seidner Co. GmbH, Riedlingen, Germany).

The specimens (160 × 40 × 40 mm prisms) were tested for bending according to a three-point scheme with a distance between the supports of Lo = 130 mm. Flexural strength fb was determined according to the known formula as the ratio between the bending moment and the moment of resistance of the cross-section. The flexural strength was determined according to the peak fracture load F_max_. In the case of fiber cement composites, the parameter of flexural strength is insufficient for the full assessment of the effect of dispersed reinforcement. The fibers continue working in the material after the crack has opened. Therefore, in this study the mid-span deflection of prisms was measured during the bending test in order to calculate the fracture work of the material.

The fracture work energy was determined according to the RILEM TC-50 FMC method. It includes elastic deformation energy and the effect of post-cracking work. Different from the requirements of the RILEM standard, no notch was made at the bottom of the specimen, since it better matches the purpose of the fibers in the material of the protective coating. The fracture energy (GF) is calculated by referring to the method recommended by RILEM in Equation (2):

(2) $$G_{F}=\frac{W_{0}+mg{\;\delta }_{max}}{A_{lig}}$$

where: *G_F_*—is the fracture energy, N/m (or Nm/m^2^); *W*_0_—is the work done by specimen bending machine measured by the load-deflection curve (area under the curve), Nm; *mg*—is the specimen weight, N; *δ_max_*—is the deflection when the residual load is 20% of the maximum load, m; *A_lig_*—is the fracture area of specimens, m^2^. It should be noted that in this case the effect of the weight of the specimens (*mgδ_max_*) has a very insignificant effect, therefore this parameter can be ignored in fractural energy calculations.

The drying shrinkage of lightweight composite was measured by a digital micrometre of 0.001 mm precision.

The amount of heat released during the hydration, and the heat release rate were measured by the calorimeter TONICAL III (Toni Technik GmbH, Berlin, Germany). The measurements were done at 20 °C temperature, the measurement duration was 48 h.

X-ray diffraction (XRD), thermal (DTG) and calorimetric analyses were done with cement paste specimens without GEG (Table 5).

The XRD analysis was performed using a DRON-7 diffractometer (Bourevestnik SJC, St. Petersburg, Russia) (with Cu-Kα (λ = 0.1541837 nm) radiation. The following test parameters were used: 30 kV voltage; 12 mA current; 2θ diffraction angle range from 4° to 60° with increment of 0.02° measured each 0.5 s. The phases present were identified comparing the XRD diffractograms with standard diffraction patterns provided by the International Centre for Diffraction Data (ICDD). The internal standard anatase was used. 9:1 (binder:anatase) specimens were prepared. The amount of compounds was valued according the intensity of the main peaks.

Thermal analysis (DTG/TG) was performed with a TGA 4000 thermal analyser (Perkin-Elmer, Waltham, MA, USA). Specimens with a mass of 50–60 mg were placed in a platinum crucible and heated at 10 °C/min in the nitrogen environment up to 700 °C. The amount of portlandite was calculated according to literature [34].

The microstructure of materials was tested with the scanning electron microscopy (SEM) device SEM JSM-7600F, JEOL, Tokyo, Japan). The following electron microscopy parameters were used: power 10 kW, distance to specimen surface from 7 to 11 mm. Characteristics of the microstructure was identified by testing the specimen splitting surface. Before testing, the splitting surface was coated with a thin electrically conductive layer of gold by evaporating the gold electrode in the vacuum using the instrument QUORUMQ150R ES (Quorum Technologies, Laughton, UK). X-ray microanalysis was performed by the energy dispersion spectrometer (EDX) Inca Energy 350 (Oxford Instruments, Abingdon, UK) using Silicon Drift Type detector X-Max20. The INCA software package (Oxford Instruments) was used.

## 3. Results and Discussion

### 3.1. Density and UPV

**Figure 2:**
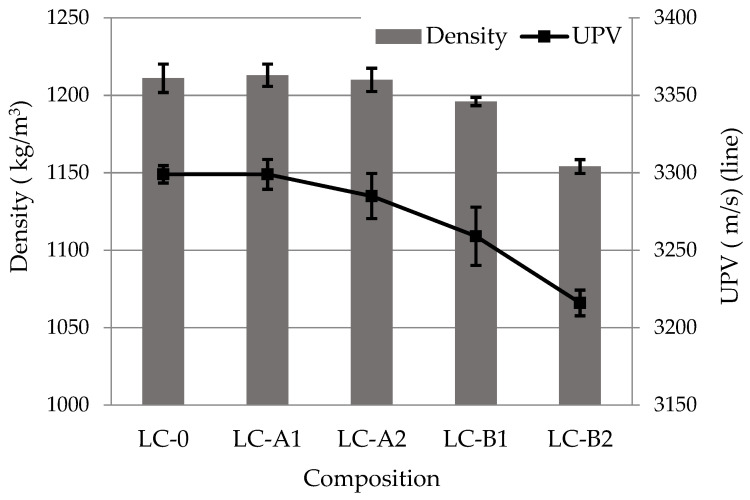
Density and UPV results in lightweight composites containing GEG after 28 days.

**Figure 3:**
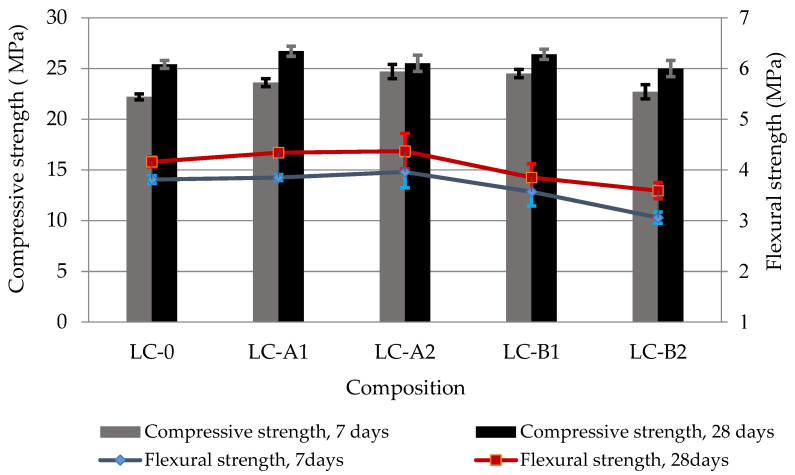
Compressive and flexural strength results of lightweight composites after 7 and 28 days of curing.

The densities of control specimens of lightweight composite containing GEG and the specimens modified with PVA fibers of Type A were almost identical whereas the specimens modified with larger fibers of Type B had a lower density. The density reduced even more (3.5%) when the fiber content was increased from 0.25% to 0.5% (Figure 2). The same downward trend was observed in UPV test results (Figure 2). It is known, that structural damages or pores disturb the propagation of ultrasonic wave and decrease its velocity; also, ultrasonic waves pass slower in crystalline structures than in amorphous structures [35]. It can be assumed, that larger PVA fibers distributed in lightweight composites with GEG create more pores between the granules, which reduces the density of the composite and the UPV (Figure 3). It was found [3] that fibers increase the amount of air entrained during their mixing, depending on the fiber size. Fiber modified pastes (especially with larger ones) are also harder to compact, and therefore the porosity of the composition may increase. In the work [3] it was determined, that the air void content of the cement paste with 0.35–1.40% PVA increased by 1–4% compared to the paste without PVA.

### 3.2. Flexural and Compressive Strength

The compressive strength of lightweight composite (Figure 3) depends very little on the amount and type of the fibers used. Only insignificant increase of compressive strength both at 7 and 28 days in the specimens with a lower fiber content (0.25%, LC-A1 and LC-B1) and strength decrease in the specimens at 28 days with a higher fiber content (0.5%, LC-A-2 and LC-B-2) was observed. In the case of a higher fiber content, the compressive strength was similar to that of the control specimen. The flexural strength of specimens with Type A fibres remained similar to that of control specimens both at 7 and 28 days, whereas in specimens containing Type B fibers the flexural strength values were lower. This indicates that it is more difficult to compact the material with lightweight round granules, such as GEG, and with Type B fibers of larger diameter.

### 3.3. Fracture Energy

**Figure 4:**
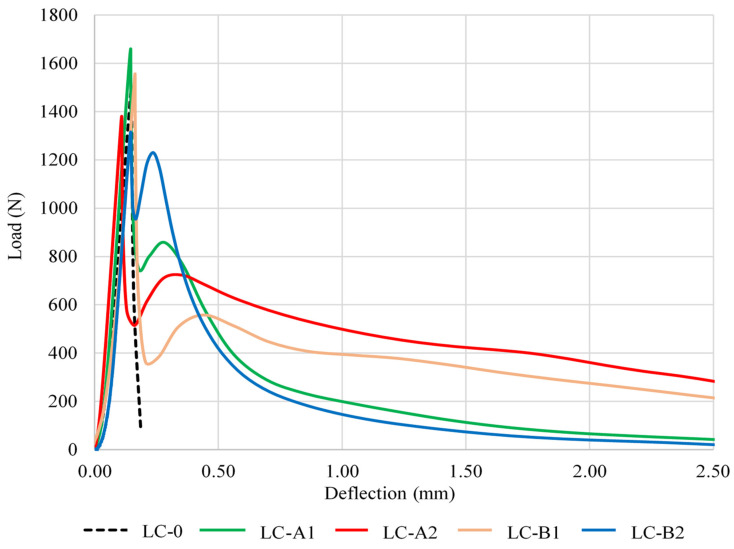
“Load–deflection” curves for specimens subjected to bending: LC-0, LC-A1, LC-A2, LC-B1, LC-B2.

All series of specimens were tested for bending according to the three-point bending test method. The most characteristic curves for each series are shown in Figure 4. Brittle fracture was observed only in the reference series LC-0 (specimens without fibers). In this case, the force decreased after reaching the maximum load and the specimen fractured along one section. In the specimens modified with PVA fibers there was a sharp drop in the load after the maximum value was reached, followed by the load stabilization phase to the residual value F_res_. To assess the performance of PVA fibers, the residual force was measured at the deflection of 0.5 and 2 mm, and the ratios F_res_/F_max_ were determined in addition. Numerical values of additional specimen bending parameters are given in Table 6. 

The analysis of the obtained results revealed that the specimens with a lower fiber content (series LC-A1 and LC-B1) had the highest peak bending load F_max_: 1649N and 1525N respectively (specimen LC-B1 showed 7.4% lower result). In turn, the specimens with a high fiber content (series LC-B2 and LC-A2) had the lowest breaking load values: 1333N and 1330N (Table 6).

The analysis of post-cracking behavior in deflection values of 0.5–2.5 mm showed that LC-A2 and LC-B1 specimens had higher values of residual load than LC-A1 and LC-B2 specimens. The comparison of fracture energy illustrated in Figure 4 and Table 6 confirm that a brittle type of degradation occurred in specimens without fibers and the energy required to destroy the specimens was close to 100 N/m. The maximum energy required to destroy the fiber modified specimens of LC-A2 series was ~ 8 times higher compared to the control specimen. The fracture energy for the specimens of LC-A1 series was ~2.5 times higher compared to the control specimen. According to literature source [36], higher fracture energies of specimens with fiber indicate that the material has a higher energy absorbing capacity compared to the control specimens. The trends reported by other researchers [37] coincide with the results obtained in our study: the fracture energy increases with the increased fiber content to a certain limit; although the fibers have a positive synergistic reinforcing effect, agglomerates may occur due to the fiber size when the content of fibers used reaches a certain limit. A higher content of larger Type B fibers (LC-B2 series) resulted in a more porous structure, which caused a decrease in the strength of lightweight composite. These specimens also had a lower fracture energy compared to LC-B1 specimens.

### 3.4. Drying Shrinkage

**Figure 5:**
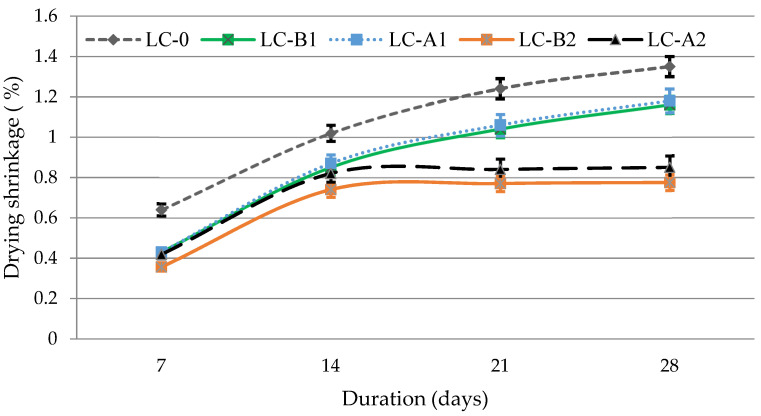
Drying shrinkage of lightweight composite while curing.

Drying shrinkage test results (Figure 5) showed that fibers of Types A and B almost identically reduce the drying shrinkage of lightweight composite while curing. With the addition of 0.25% of PVA fibers of both types the shrinkage of the lightweight composite reduced evenly by approximately 13%. With the increase of the fiber content up to 0.5% the shrinkage pattern was similar to the shrinkage with a lower fiber content until 14 day of curing. The effect of the higher fiber content became apparent after 14 days of curing. After 28 days the shrinkage of the composite with Type A fibers decreased ~28% and with Type B fibers ~34% compared to the composites containing 0.25% of the fibers. Compared to the control specimen the shrinkage of the composite with Type A fibers decreased ~37% and with B Type fibers ~42%. Obviously the addition of fibers can significantly reduce the drying shrinkage of lightweight composite modified with GEG.

### 3.5. Calorimetry

**Figure 6:**
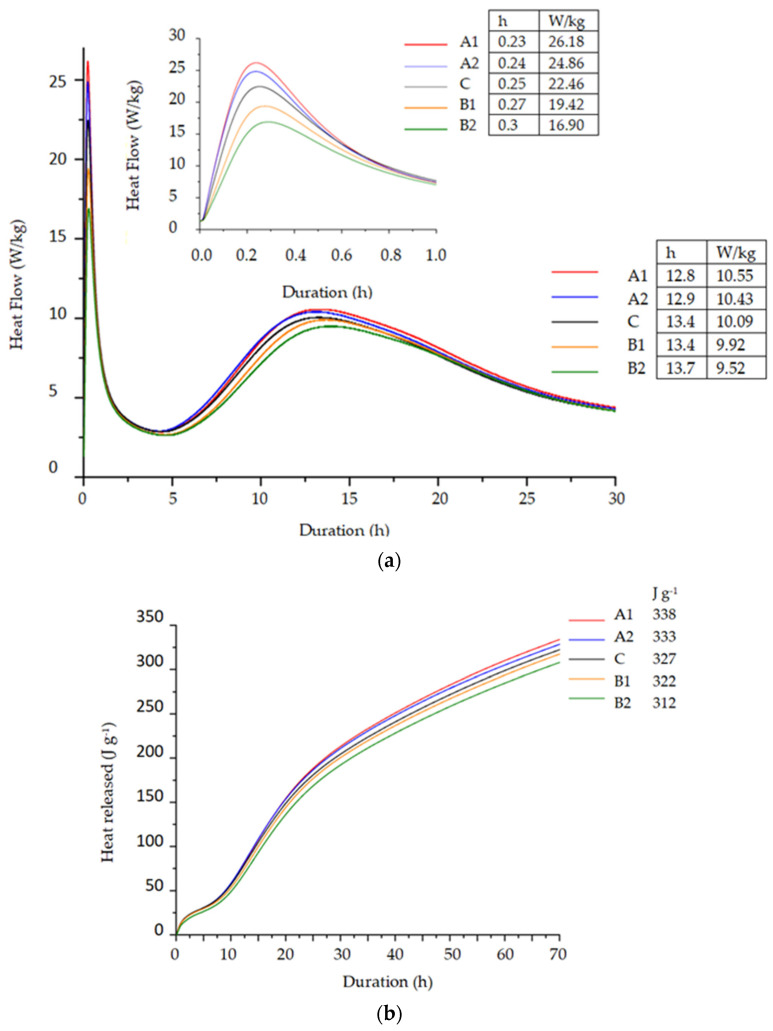
Heat release rate (**a**) and amount of released heat (**b**) during 70 h of hydration of blended cement pastes with PVA fibers.

The crystallization stage of cement hydration in mixtures containing PVA fibers of Type A was faster (the maximum of it was reached after 12.8 h or 12.9 h) than in control specimens without fibers (13.4 h) (Figure 6a). The process in specimens containing 0.25% of Type B fibers was similar to the control specimen and in specimens containing 0.5 % of fibers it was slightly slower (13.7 h). The results of the total heat released (Figure 6b) show that the lowest amount of heat (312–322 J/g) was released in specimens containing Type B fibers, which are larger and have a smaller surface area in the mix compared to PVA fibers of Type A. The specimens modified with 0.25% of Type A fibers released about 10 J/g more heat than control specimens. The total heat released by specimens with a higher content of PVA fibers of Type A (0.5%) was almost the same as in control specimens. Hydrophilic fibers of Type A with a rather big surface area are supposed to change the pattern of water movement in the cement matrix [10,11,12] thus activating hydration at the surface of the fibers. Apparently this characteristic is reflected in the higher heat release in the cement paste during the first 20 min after mixing the composition with water (Figure 6a, left corner), due to the better wetting effect of the cement particles and the initial reactions (formation of ettringite).

### 3.6. XRD Analysis

**Figure 7:**
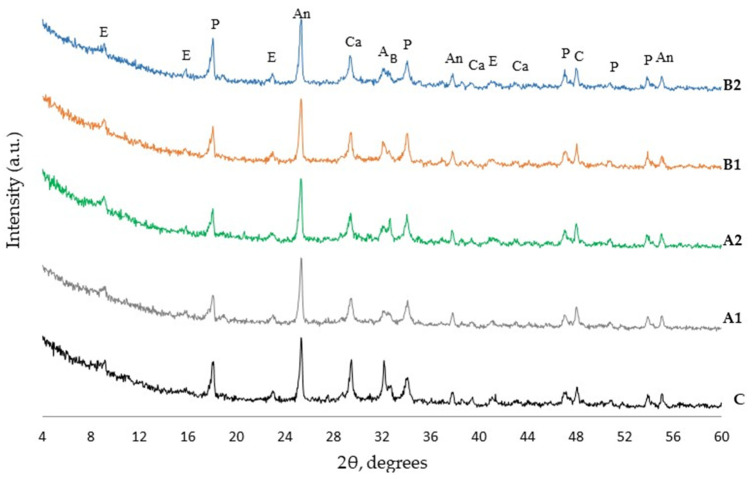
XRD diffractogram of hardened blended cement paste after 7 days curing (E—ettringite, P—portlandite, An—anatase (internal standard), A—alite, B—belite, Ca—calcite).

**Figure 8:**
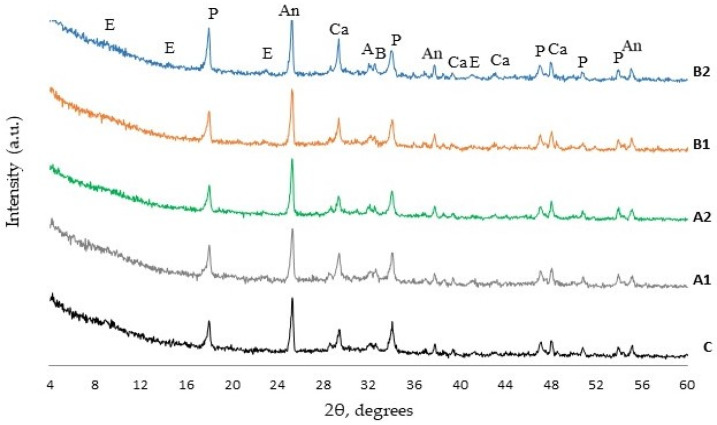
XRD diffractogram of hardened blended cement paste after 28 days curing (E—ettringite, P—portlandite, An—anatase (internal standard), A—alite, B—belite, C—calcite).

**Figure 9:**
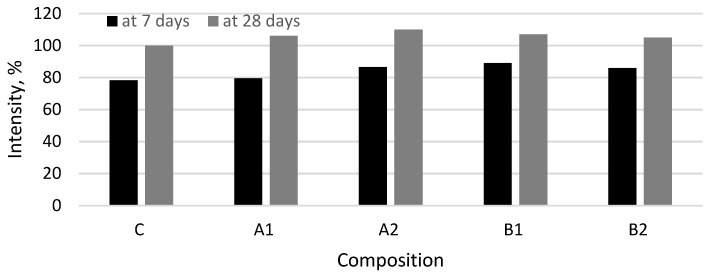
Semi-quantification of portlandite present is the samples assuming as reference for 100% content of the C paste at 28 days.

The results of XRD analysis after 7 and 28 days of curing (Figure 7 and Figure 8) showed the presence of the same minerals—ettringite, portlandite, calcite, alite, belite, and anatase used as internal standard—in all cement paste compositions. The relative evaluation of mineral content according to the intensity of their main peaks revealed that the intensity of the main peak of portlandite (d = 0.263 nm) in fiber modified specimens was higher (Figure 9). The highest intensity of portlandite at 7 and 28 days of curing was found in specimens of compositions A2 and B1. After 28 days the highest intensity was observed in specimens of composition A2 showing a difference of 10% compared to the control specimen. The addition of PVA fibers to the mix also caused a significant drop of the main peaks of alite (d = 0.277 nm) at 7 days. These results show, that during the first hydration days PVA accelerate cement hydration, but at 28 days the amount of cement minerals in all compositions is quite similar, only the amount of portlandite in compositions with PVA is higher and the amount of ettringite is lower. The tendencies of XRD analysis results suggest that PVA fibers promote cement hydration especially, in early hydration period.

### 3.7. Thermal Analysis

**Figure 10:**
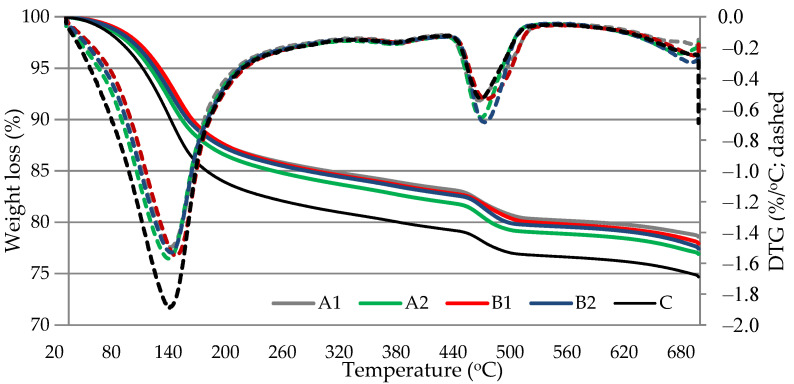
Results of thermal analysis of hardened blended cement paste after 7 days curing.

**Figure 11:**
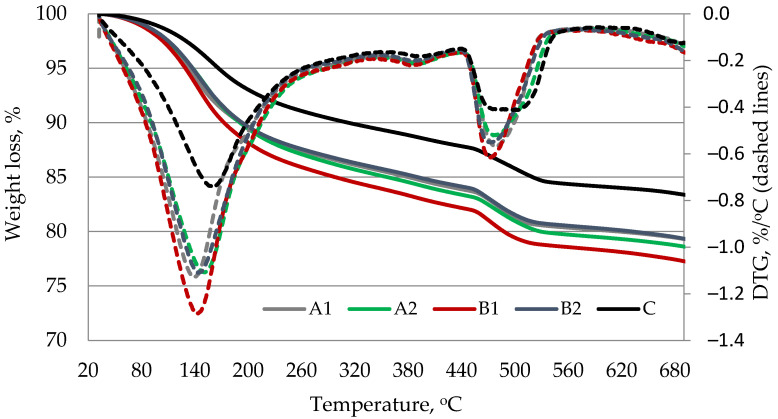
Results of thermal analysis of hardened blended cement paste after 28 days curing.

DTG/TG analysis results (Figure 10 and Figure 11) showed two main endo-effects when heating the blended cement paste specimens at up to 700 °C temperature. The first endo peak formed in the temperature range between 30 °C and ~230 °C. It is known that free water separation and decomposition of ettringite and CSH occur in this temperature range. The second peak attributed to the decomposition of portlandite was recorded in the temperature range between ~430 °C and ~560 °C. According to literature references [38], the most of evaporable water is removed in the range of 30–105 °C, decomposition of ettringite, the loss of a portion of water of CASH and CSH, and the removal of all evaporable water occurs in the range of 110–170 °C, the loss of the remaining part of CSH, especially CASH, occurs at 180–350 °C, and the dihydroxylation of portlandite occurs at ~430–560 °C. The results showed (Table 7) that according to the mass loss in 430–560 °C temperature range, the PVA fibers support the increase of portlandite content in hardened cement paste after 7 and 28 days of curing. This compound is able to fill the pores, thus contributing to the reduction of porosity and restrict the volumetric changes associated with CSH, thus contributing to the reduction of shrinkage [39]. Moreover, after 28 days a higher weight loss than in control paste was observed in 110–170 °C and 180–350 °C temperature ranges in PVA modified specimens indicating that higher amounts of such hydration products as CSH and CASH were formed in those specimens. The higher degree of cement hydration in the presence of fibers may be one of the reasons why the compressive strength of certain composite with GEG are higher than those of the control composite (Figure 3).

### 3.8. SEM/EDX Analysis

**Figure 12:**
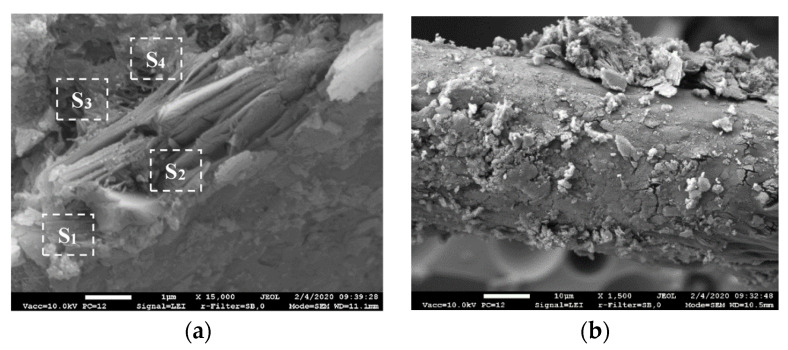
Characteristic microstructure of hardened blended cement paste: S1–S4 the area of EDX analysis (**a**) and pulled out PVA fiber (**b**).

The SEM analysis of the cement paste after 28 days of curing revealed the formation of a dense layer of 2–3 µm sized densified hydrate zone around PVA fibers (Figure 12a). 

Well adhered particles of the cement matrix are seen on the surface of the fiber pulled out from the composite (Figure 12b). It proves good adhesion of the PVA fiber and the hardened cement paste ensured by the rough surface of the fiber with grooves (Figure 1).

**Figure 13:**
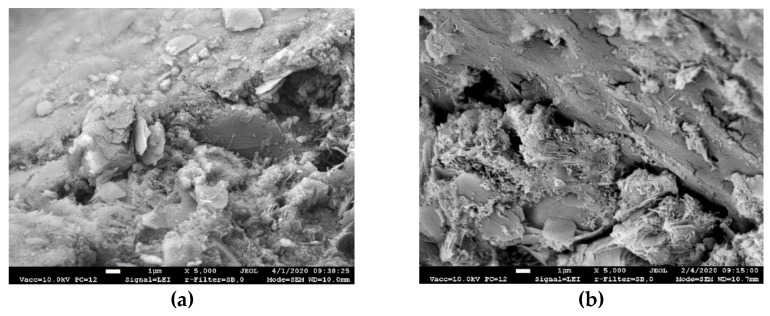
Microstructure of hardened blended cement paste (**a**) with Type A fibers; (**b**) with Type B fibers.

EDX analysis of the characteristic densified zone of hydrate around the PVA fiber (Table 8) showed the presence of the highest amounts of calcium and oxygen, silicon and very small amount of aluminum. It can be said that portlandite and CSH, and a small amount of CASH are mostly formed in this area.The increased amount of silicon, oxygen and decreased amount of calcium in the area behind the dense layer (S3 and S4) indicate different compositions of hydrates in the hardened cement paste and in the layer formed around the fiber. Heinz et al. noted [40] that a higher amount of portlandite is formed around the aggregates (and visible around the fibers in our case). As a combined pozzolanic additive was used in the work, more CSH and CASH especially are formed in the hardened cement paste behind the formed layer. These research results (identification of densified hydrate zones around the fibers) supplement our explanation of the influence of PVA fibers on the cement hydration and subesquent improvement of mechanical properties and the decrease of shrinkage of the composite with GEG. A higher amount of fibres (particularly of Type B, Figure 13) increases the amount of small air voids, which could lead to decreasing of density, UPV and flexural strength values of lightweight composite with GEG (Figure 2 and Figure 3).

## 4. Conclusions

PVA fibers (Type A: Ø40 µm, L = 8 mm and Type B: Ø200 µm, L = 12 mm) have strong adhesion with the cement matrix due to densified hydrate zones formed around the fibers. A higher amount of portlandite is clearly visible in those areas, especially in the case of specimens with Type A fibers. XRD analysis showed higher amounts of portlandite in specimens with PVA fibers. DTG analysis confirmed that specimens with PVA fibers had more portlandite formed. A higher mass loss in 180–350 °C temperature range after 28 days suggests that more CSH and CASH were formed in specimens with PVA fibers. Calorimetry analysis results revealed that Type A fibers accelerate cement hydration while larger fibers of Type B have no significant effect on hydration time.

PVA fibers (Types A and B) make it possible to reduce the drying shrinkage of lightweight composite modified with GEG by approximately 40% after 28 days of curing. The drying shrinkage decreased due to a strong physical link between the fiber and the cement matrix and higher amount of portlandite. PVA fibers of Type A had a more significant positive effect on the improvement of physical and mechanical properties as well as the structure of the cement matrix in lightweight composite modified with GEG, because of a larger surface area. Specimens with Type A fibers have higher values of ultrasonic pulse velocity. This also indicates the formation of a denser structure of the cement matrix and a denser interfacial contact zone.

The results of the physical and chemical analysis of the specimens were confirmed by the results of the testing of mechanical and deformation properties of the material. The specimens with fibers require a significantly higher fracture energy to destroy them. Type A fibers added at 0.5% were more effective in terms of bending and deformation resistance. Compared to the control specimens, the fracture energy increased about eight times. Larger size fibers of Type B tend to agglomerate when added at 0.5% resulting in a more porous structure, lower strength, and lower fracture energy of the lightweight composite. LC-A1 and LC-B1 specimens with a lower fiber content showed a much higher load-bearing capacity.

## Figures and Tables

**Table 1:** Chemical composition of cement (%).

Oxides (%)	CaO	SiO_2_	Al_2_O_3_	Fe_2_O_3_	MgO	K_2_O	Na_2_O	SO_3_	Cl	L.O.I.
Cement	63.2	20.4	4.0	3.6	2.4	0.9	0.2	3.1	0.05	2.15

**Table 2:** Chemical composition of FCCCW and MW (%).

Oxides (%)	SiO_2_	Al_2_O_3_	Fe_2_O_3_	MgO	K_2_O	Na_2_O	SO_x_	CaO	Mn_2_O_3_	TiO_2_	Others
FCCCW	50.1	41.3	1.3	0.49	0.07	0.2	2.3	0.5	0.06	-	1.9
MW	54.3	34.0	1.14	0.51	0.80	3.26	0.15	1.94	-	0.53	3.37

**Table 3:** The characteristics of GEG.

Characteristics	Size of GEG (mm)		
	1.0–2.0	0.5–1.0	0.25–0.5
Bulk density (kg/m^3^)	230.0	270.0	340.0
Compressive strength (MPa)	2.0	2.3	2.5
Water absorption (%) by mass	20.0	20.0	25.0
pH	10.6		
Softening point (°C)	~700		

**Table 4:** Characteristics of PVA fibers.

Type of Fibers	Diameter (µm)	Length (mm)	Tensile Strength (GPa)	Elasticity Modulus (GPa)
A	40	8	1.6	42
B	200	12	1.0	30

**Table 5:** Compositions of cement paste and lightweight composite (% by mass).

Mark of Compositions	PC	FCCCW	MW	PVA-A *	PVA-B *	GEG				
						0.25–0.5 (mm)	0.5–1.0 (mm)	1.0–2.0 (mm)	SP *	W/B
C	90	5	5						1	0.35
A1	90	5	5	0.25					1	0.35
A2	90	5	5	0.5					1	0.35
B1	90	5	5		0.25				1	0.35
B2	90	5	5		0.5				1	0.35
LC-0	63	3.5	3.5			10	10	10	1	0.4
LC-A1	63	3.5	3.5	0.25		10	10	10	1	0.4
LC-A2	63	3.5	3.5	0.5		10	10	10	1	0.4
LC-B1	63	3.5	3.5		0.25	10	10	10	1	0.4
LC-B2	63	3.5	3.5		0.5	10	10	10	1	0.4

* more than 100%.

**Table 6:** Bending characteristics values and bending energy for tested specimens.

Composition	F_max_ (N)	F_res_ (N)	F_res_/F_max_	F_0.5_ (N)	F_0.5_/F_max_	F_1.5_ (N)	F_1.5_/F_max_	G_f_ (N/m)
LC-0	1646	0	0.00	0	0.00	0	0.00	100
LC-A1	1649	852	0.52	366	0.22	102	0.06	259
LC-A2	1330	722	0.54	665	0.50	434	0.33	830
LC-B1	1525	524	0.34	503	0.33	324	0.21	497
LC-B2	1333	1271	0.95	444	0.33	84	0.06	317

**Table 7:** Weight loss of hardened cement paste specimens in different temperature intervals (%).

Mark of Composition	110–170 °C	180–350 °C	430–560 °C	Amount of Portlan-Dite, %
after 7 days				
C	9.6	4.5	2.7	14.7
A1	7.7	4.1	3.1	16.0
A2	8.0	4.4	3.1	16.3
B1	7.9	4.3	3.2	16.7
B2	7.8	4.3	3.1	16.2
after 28 days				
C	3.7	4.7	3.6	17.7
A1	5.9	5.1	3.7	18.9
A2	5.9	5.6	3.8	19.8
B1	6.8	5.5	3.8	19.7
B2	5.9	5.1	3.8	19.2

**Table 8:** The element composition of sample with PVA fibers of Type A.

Marking of Tested Zone	Chemical Element (%)			
	Ca	O	Si	Al
S_1_	75.4	22.4	1.8	0.4
S_2_	72.2	24.2	3.3	0.3
S_3_	59.9	31.7	7.5	0.9
S_4_	54.1	37.2	7.9	0.8

## Data Availability

Not applicable.

## References

[1] Thong C.C., Teo D.C.L., Ng C.K. (2016). Application of polyvinyl alcohol (PVA) in cement-based composite materials: A review of its engineering properties and microstructure behavior. Constr. Build. Mater..

[2] Xu B., Toutanji H.A., Lavin T., Gilbert J.A. (2011). Characterization of polyvinyl alcohol fiber reinforced organic aggregate cementitious materials. Key Eng. Mater..

[3] Kim J.H., Robertson R.E. (1998). Effects of polyvinyl alcohol on aggregate-paste bond strength and the interfacial transition zone. Adv. Cem. Based Mater..

[4] Arisoy B., Wu H. (2008). Material characteristics of high performance lightweight concrete reinforced with PVA. Constr. Build. Mater..

[5] Bentur A., Mindess S., Skalny J. (1989). Fiber Reinforced Cements and Concretes Recent Developments.

[6] Fahad A.M., Mingxue W., Jianyong C., Huapeng Z. (2019). Study on PVA fiber surface modification for strain-hardening cementitious composites (PVA-SHCC). Constr. Build. Mater..

[7] Haque M.A., Chen B., Ahmad M.R., Farasat Ali Shah S. (2020). Mechanical strength and flexural parameters analysis of micro-steel, polyvinyl and basalt fiber reinforced magnesium phosphate cement mortars. Constr. Build. Mater..

[8] Li Z., Perez Lara M.A., Bolander J.E. (2006). Restraining effects of fibers during non-uniform drying of cement composites. Constr. Build. Mater..

[9] Zhang J., Li V.C. (2001). Influences of Fibers on Drying Shrinkage of Fiber-Reinforced Cementitious Composite. J. Eng. Mech..

[10] Antonovič V., Sikarskas D., Malaiškienė J., Boris R., Stonys R. (2019). Effect of pozzolanic waste materials on hydration peculiarities of portland cement and granulated expanded glass- based plaster. J. Therm. Anal. Calorim..

[11] Qiu J., Lim X.N., Yang E.H. (2016). Fatigue-induced deterioration of the interface between micro-polyvinyl alcohol (PVA) fiber and cement matrix. Cem. Concr. Res..

[12] Li Y., Li W., Deng D., Wang K., Duan W.H. (2018). Reinforcement effects of polyvinyl alcohol and polypropylene fibers on flexural behaviors of sulfoaluminate cement matrices. Cem. Concr. Compos..

[13] Yang E.H., Wang S., Yang Y., Li V.C. (2008). Fiber-Bridging Constitutive Law of Engineered Cementitious Composites. J. Adv. Concr. Technol..

[14] Qiu J., Lim X.N., Yang E.H. (2017). Fatigue-induced in-situ strength deterioration of micro-polyvinyl alcohol (PVA) fiber in cement matrix. Cem. Concr. Compos..

[15] Yu J., Zhang M., Li G., Meng J., Leung C.K.Y. (2020). Using nano-silica to improve mechanical and fracture properties of fiber-reinforced high-volume fly ash cement mortar. Constr. Build. Mater..

[16] Ercikdi B., Cihangir F., Kesimal A., Deveci H., Alp İ. (2009). Utilization of industrial waste products as pozzolanic material in cemented paste backfill of high sulphide mill tailings. J. Hazard. Mater..

[17] Pacewska B., Bukowska M., Wilińska I., Swat M. (2002). Modification of the properties of concrete by a new pozzolan—A waste catalyst from the catalytic process in a fluidized bed. Cem. Concr. Compos..

[18] Torres M.L., García-Ruiz P.A. (2009). Light weight pozzolanic materials used in mortars: Evaluation of their influence on density, mechanical strength and water absorption. Cem. Concr. Compos..

[19] Khandaker M., Hossain A. (2004). Properties of volcanic pumice based cement in lightweight concretes. Cem. Concr. Res..

[20] Vaganov V., Popov M., Korjakins A., Šahmenko G. (2017). Effect of CNT on Microstructure and Minearological Composition of Lightweight Concrete with Granulated Foam Glass. Procedia Eng..

[21] Güneyisi E., Gesoğlu M., Özbay E. (2010). Strength and drying shrinkage properties of self-compacting concretes incorporating multi-system blended mineral admixtures. Constr. Build. Mater..

[22] Pacewska B., Wilinska I., Bukowska M., Nocun-Wczelik W. (2002). Effect of waste aluminosilicate material on cement hydration and properties of cement mortars. Cem. Concr. Res..

[23] Paya J., Monzo J., Borrachero M.V., Velázquez S. (2003). Evaluation of the pozzolanic activity of fluid catalytic cracking catalyst residue (FC3R). thermogravimetric analysis studies on FC3R-portland cement pastes. Constr. Build. Mater..

[24] Paya J., Monzo J., Borrachero M.V. (1999). Fluid catalytic cracking catalyst residue (FC3R) an excellent mineral by- product for improving early-strength development of cement mixtures. Cem. Concr. Res..

[25] Pacewska B., Wilińska I., Bukowska M., Blonkowski G., Nocuń-Wczelik W. (2004). An attempt to improve the pozzolanic activity of waste aluminosilicate catalyst. J. Therm. Anal. Calorim..

[26] Šeputytė-Jucikė J., Kligys M., Sinica M. (2016). The effects of modifying additives and chemical admixtures on the properties of porous fresh and hardened cement paste. Constr. Build. Mater..

[27] Vejmelkova E., Pavlikova M., Keppert M., Kersner Z., Rovnanikova P., Ondracek M. (2010). High performance concrete with Czech metakaolin: Experimental analysis of strength, toughness and durability characteristics. Constr. Build. Mater..

[28] Mastali M., Kinnunen P., Isomoisio H., Karhu M., Illikainen M. (2018). Mechanical and acoustic properties of fiber-reinforced alkali-activated slag foam concretes containing lightweight structural aggregates. Constr. Build. Mater..

[29] Mueller A., Sokolova S., Vereshagin V. (2008). Characteristic of lightweight aggregates from primary and recycled raw materials. Constr. Build. Mater..

[30] Bumanis G., Bajare D., Korjakins A. (2013). Mechanical and thermal properties of lightweight concrete made from expanded glass. J. Sustain. Archit. Civil Eng..

[31] Adhikary S.K., Ashish D.K., Rudžionis Ž. (2021). Expanded glass as light-weight aggregate in concrete—A review. J. Clean. Prod..

[32] Gorospe K., Booya E., Ghaednia H., Das S. (2019). Effect of various glass aggregates on the shrinkage and expansion of cement mortar. Constr. Build. Mater..

[33] Limbachiya M.C., Meddah M.S., Fotiadou S. (2012). Performance of granulated foam glass concrete. Constr. Build. Mater..

[34] Malaiskiene J., Costa C., Baneviciene V., Antonovic V., Vaiciene M. (2021). The effect of nano SiO_2_ and spent fluid catalytic cracking catalyst on cement hydration and physical mechanical properties. Constr. Build. Mater..

[35] Xu J., Wei H. (2019). Ultrasonic Testing Analysis of Concrete Structure Based on S Transform. Shock Vib..

[36] Mikhailenko P., Cassagnabere F., Emam A., Lachemi M. (2018). Influence of physico-chemical characteristics on the carbonation of cement paste at high reploacement rates of metakaolin. Constr. Build. Mater..

[37] Shi X., Brescia-Norambuena L., Tavares C., Grasley Z. (2020). Semicircular bending fracture test to evaluate fracture properties and ductility of cement mortar reinforced by scrap tire recycled steel fiber. Eng. Fract. Mech..

[38] Yang Y., Zhou Q., Deng Y., Lin J. (2020). Reinforcement effects of multi-scale hybrid fiber on flexural and fracture behaviors of ultra-low-weight foamed cement-based composites. Cem. Concr. Compos..

[39] Costa C., Goncalves M.C., Margarido F. (2015). Hydraulic binders. Materials for Construction and Civil Engineering Science.

[40] Heinz D., Urbonas L., Gerlicher T. (2012). Effect of Heat Treatment Method on the Properties of UHPC. Ultra-High Performance Concrete and Nanotechnology in Construction. Proceedings of Hipermat 2012 3rd International Symposium on UHPC and Nanotechnology for high Performance construction Materials.

